# TruNeo: an integrated pipeline improves personalized true tumor neoantigen identification

**DOI:** 10.1186/s12859-020-03869-9

**Published:** 2020-11-18

**Authors:** Yunxia Tang, Yu Wang, Jiaqian Wang, Miao Li, Linmin Peng, Guochao Wei, Yixing Zhang, Jin Li, Zhibo Gao

**Affiliations:** 1 YuceBio, 2002#, ShenYan Road, Dabaihui Center, Yantian distict, Shenzhen, 518020 China; 2 Yutai Antigen Science, Building A28, Life Science Park, 140 Jinye Road, Dapeng New District, Shenzhen, 518000 China; 3 BGI Education Center, University of Chinese Academy of Sciences, Shenzhen, 518083 China; 4 Cancer Research Institute of Yucebio, 2002#, ShenYan Road, Dabaihui Center, Yantian distict, Shenzhen, 518020 China; 5 Department of Pulmonary and Critical Care Medicine, The Seventh Affiliated Hospital, Sun Yat-Sen University, Shenzhen, 518107 China

**Keywords:** Neoantigen, Multiple factors, Recall rate, Positive rate, Top-ranked

## Abstract

**Background:**

Neoantigen-based personal vaccines and adoptive T cell immunotherapy have shown high efficacy as a cancer treatment in clinical trials. Algorithms for the accurate prediction of neoantigens have played a pivotal role in such studies. Some existing bioinformatics methods, such as MHCflurry and NetMHCpan, identify neoantigens mainly through the prediction of peptide-MHC binding affinity. However, the predictive accuracy of immunogenicity of these methods has been shown to be low. Thus, a ranking algorithm to select highly immunogenic neoantigens of patients is needed urgently in research and clinical practice.

**Results:**

We develop TruNeo, an integrated computational pipeline to identify and select highly immunogenic neoantigens based on multiple biological processes. The performance of TruNeo and other algorithms were compared based on data from published literature as well as raw data from a lung cancer patient. Recall rate of immunogenic ones among the top 10-ranked neoantigens were compared based on the published combined data set. Recall rate of TruNeo was 52.63%, which was 2.5 times higher than that predicted by MHCflurry (21.05%), and 2 times higher than NetMHCpan 4 (26.32%). Furthermore, the positive rate of top 10-ranked neoantigens for the lung cancer patient were compared, showing a 50% positive rate identified by TruNeo, which was 2.5 times higher than that predicted by MHCflurry (20%).

**Conclusions:**

TruNeo, which considers multiple biological processes rather than peptide-MHC binding affinity prediction only, provides prioritization of candidate neoantigens with high immunogenicity for neoantigen-targeting personalized immunotherapies.

## Background

Neoantigens are tumor-specific antigens formed by somatic mutations and are ideal targets for immunotherapy. They are highly immunogenic because they are not expressed in normal tissues and hence bypass central thymic tolerance. In humans, effective antitumor immunity has been associated with the presence of T cells directed at neoantigens [1] and in recent years, neoantigen-based personal vaccines and adoptive T cell immunotherapies have shown strong therapeutic effects on cholangiocarcinoma, colorectal cancer, breast cancer, melanoma and glioma [2–7]. Moreover, neoantigens have also been shown to be strong targets for more established immune checkpoint blockade therapies [8].

Next-generation sequencing data has been widely applied to predict neoantigens, and a number of bioinformatic tools have already been developed. For example, NetMHCpan [9] and MHCflurry [10] can predict the ability of mutant peptides to bind to class I or class II HLAs. These tools achieve good performance as predictors of binding affinity but poor performance as predictors of actual HLA presentation, let alone immunogenicity, or T cell response against neoantigens. Previous studies reported that less than 5% of neoantigens identified using these methods can be successfully found on the surfaces of tumor cells. The in-silico neoantigen prediction models based mainly on MHC binding affinity are limited by low predictive performance for actual immunogenicity, likely because these models considered one of the multiple steps in the neoantigen presentation processes while ignoring other steps, hence potentially underestimating the complexity of forming of true immunogenic neoantigens. The typical pipeline for the identification of immune-relevant neoantigens consists of six main steps [11].Therefore, it is necessary to add more biological factors to these prediction algorithms. We have developed an integrated pipeline called TruNeo to predict neoantigens by considering the following biological factors: peptide-MHC class I binding affinity, proteasomal C terminal cleavage, transporter associated with antigen processing (TAP) transport efficiency, expression abundance, tumor heterogeneity, clonality and HLA LOH (loss of heterozygosity).

Immunotherapies are partially limited in the number of specific antigens that can be targeted. In clinical trials [2, 5, 12], the top-ranked 10–20 mutations predicted by bioinformatic tools are incorporated into neoantigen-based personal vaccines. Thus patients can benefit if more immunogenic neoantigens of greater immunogenicity are included in the top-ranked predicted neoantigens. The aim of our study was to improve the positive immunogenicity rate of top-ranked predicted neoantigens, so we compared the predictive performance of TruNeo and other algorithms according to this evaluation criterion. We compared several predictive methods by counting the number of immunogenic neoantigens in the top-ranked 5, 10 or 20 mutations of 13 patients from published data. We also compared the top 10 personalized neoantigens in a lung cancer patient as predicted by TruNeo and MHCflurry. We show that the integrated TruNeo pipeline model improves personalized true tumor neoantigen identification.

## Methods

### TruNeo pipeline

The TruNeo pipeline required two data types as input; raw DNA sequencing FASTQ files from the paired tumor and normal samples, and RNA-seq data from a tumor sample. In the first step annotated somatic mutation information, HLA genotype and gene expression information were prepared. Then, the candidate neoantigens were predicted based on peptide-MHC binding affinity. In the third step, candidate neoantigens were scored by integrating information from multiple neoantigen presentation processes. Lastly, high confidence neoantigens were filtered and output.

### Generation of annotated mutation information, HLA genotype and gene expression information

Raw sequencing data of DNA from paired tumor and normal samples were aligned to a reference genome for generating bam files, and then somatic single nucleotide variants (SNVs) and InDels were identified and annotated. Normal bam was used for HLA genotyping. RNA FASTQ file from tumor samples were used for fusion identification and gene expression quantification.

#### Identification and annotation of somatic SNVs and InDels

Paired-end reads were aligned to the NCBI human reference genome (hg19) using BWA (v0.7.12) [13] with the default parameters. Picard (v1.134) (https://picard.sourceforge.net/) was used to identify duplicates, and then the Genome Analysis Toolkit (v3.3, GATK IndelRealigner) [14] was used to improve the alignment accuracy.

Somatic SNVs were detected by VarScan (v2.4.1) [15], with high confidence SNVs filtered according to the following criteria: (1) depth at mutation position ≥ 10×, and variant allele fraction (VAF) ≥ 5% in tumor and < 2% in normal; (2) distance between adjacent SNVs > 10 bp; (3) mapping quality of the mutant allele ≥ 30 (Wilcoxon rank sum test, *p* < 0.2); (4) base quality of the mutant allele ≥ 20 (Wilcoxon rank sum test, *p* < 0.05); (5) mutations not enriched within 5 bp of the 5′ or 3′ end of the read (Wilcoxon rank sum test, *p* < 0.1).

The GATK Somatic InDel Detector (v3.3, GATK IndelRealigner) was used to identify somatic insertions and deletions (InDels) with the default parameters. InDels with high confidence were filtered using the following steps: (1) local realignment was performed with combined normal and tumor BAM files for each predicted somatic InDel; (2) depth at mutation position ≥ 10×, VAF of InDels > 10% in tumor and < 2% in normal.

Finally, all SNVs and InDels were annotated using an in-house annotation software based on snpeff [16].

#### Genotyping and loss of heterozygosity in HLA class I genes

The HLA genotype was identified with the combined use of Polysover (v1.0) [17] and BWA-HLA (v1.3). If Polysover identified the same genotype in both tumor and normal samples, Polysover’s result was taken as the HLA genotype. If not, the result of BWA-HLA was checked. If BWA-HLA identified the same genotype across tumor and normal samples, the BWA-HLA result was used. If not, both the result of Polysover and BWA-HLA were considered. If Polysover and BWA-HLA identified the same genotype in the normal sample, the normal HLA genotype result was used. If Polysover and BWA-HLA were not in agreement, Polysover’s result in the normal sample was taken as the HLA genotype and marked as low confidence.

The tumor and matched normal sequences were mapped to the HLA reference, and an HLA-LOH event was reported if paired *t* test of HLA allelic imbalance was significant (*p* value < 0.0002). The allelic imbalance is tested using the ratio of log2 (Tumor unique reads/Normal unique reads) [18].

#### Fusion identification and gene expression quantification from RNA sequencing data

The raw RNA-seq data were processed with STAR v2.5.3a [19], and the gene expression level was estimated as the transcripts per million (TPM) via RSEM v1.3.0 [20]. In addition, RNA-based gene fusion was detected by STAR fusion [21], which provided another source of neoantigens.

### Prediction of candidate neoantigens based on peptide-MHC binding affinity

21-mer polypeptides centred on mutated residues were scanned to identify candidate peptides binding to class I HLAs, such as peptide sequences surrounding mutated amino acids resulting from missense mutations and frame-shift or non-frame-shift InDels. The binding affinity of 8–11-mer peptides for class I HLAs was predicted using the NetMHCPan 3.0 [22] binding algorithm. Epitopes were filtered if the following conditions were met: (1) mutations were not expressed according to RNA-seq data (mutations with mutant allele reads ≥ 1 in RNA sequencing data [23] were confirmed as expressed); (2) the sequence was homologous to self; (3) the half-maximum inhibitory concentration (IC50) according to NetMHCPan 3.0 was larger than 500 nM.

### Scoring of candidate neoantigens by integrating information from multiple neoantigen presentation processes

We first combined biological processes including MHC binding, proteasomal cleavage efficiency and TAP transport efficiency, and then integrated variant allele frequency, expression abundance and type of neoantigen. Thus, the final score for each neoantigen was calculated as follows:

$${\mathbf{PeptideScore}}\left( {\text{p}} \right) = { }{\mathbf{CombineScore}}\left( {\text{p}} \right) \cdot {\mathbf{ExpressionScore}}\left( {\text{p}} \right) \cdot {\mathbf{VAF}}\left( {\text{p}} \right) \cdot {\mathbf{PeptideWeight}}\left( {\text{p}} \right)$$

$$\begin{aligned} {\mathbf{CombineScore}}\left( {\text{p}} \right) & = 0.8{\mathbf{MHCBindingScore}}\left( {\text{p}} \right) + 0.15{\mathbf{ProteasomalCleavageScore}}\left( {\text{p}} \right) \\ & \quad + \,0.05{\mathbf{TAPTransportScore}}\left( {\text{p}} \right) \\ \end{aligned}$$

$${\mathbf{MHCBindingScore}}\left( {\text{p}} \right) = {\tanh}\left( {\left( {{5}00 - {\text{MHCBindingAffinity}}\left( {\text{p}} \right)} \right)/{2}00} \right)$$

$${\mathbf{TAPTransportScore}}\left( {\text{p}} \right) = {\tanh}({\text{TAPPrediction}}\left( {\text{p}} \right)*{2}.{5})/{2} + 0.{5}$$

$${\mathbf{ProteasomalCleavageScore}}\left( {\text{p}} \right) = {\tanh}({\text{CleavagePrediction}}\left( {\text{p}} \right)*{3})$$

The proteasomal cleavage efficiency was predicted by netChop [24], and the TAP transport efficiency was predicted by netCTLpan [25].

Expression score was identified by TPM, and normalised based on ranking status.

$${\mathbf{Expression}} {\mathbf{Score}}\left( {\text{p}} \right) = \left\{ {\begin{array}{*{20}l} {1,\quad if \;TPM > upper \;quartile} \hfill \\ {0.66, \quad if\; lower \;quartile < TPM < upper \;quartile} \hfill \\ {0.33, \quad if \;TPM < lower\; quartile} \hfill \\ {0,\quad TPM = 0} \hfill \\ \end{array} } \right.$$

$${\mathbf{PeptideWeight}}\left( {\text{p}} \right) = {\mathbf{NeoantigenTypeWeight}}\left( {\text{ p}} \right) \cdot {\mathbf{DeepLearningWeight}}\left( {\text{p}} \right)$$

Previous studies have found that different types of neoantigen have different immunogenicity levels, which will influence their utility for vaccine selection. Epitopes were divided into 6 classes. Class1: neoORFs with high predicted affinity (< 150 nM); Class2: somatic single nucleotide variations caused by anchor residue changes with high predicted affinity (< 150 nM); Class3: somatic single nucleotide variations caused neither by neoPRFs nor anchor residue changes with high predicted affinity (< 150 nM); Class4: neoORFs with epitopes with low binding affinity (150–500 nM); Class5: somatic single nucleotide variations caused by anchor residue changes with low binding affinity (150–500 nM); Class6: somatic single nucleotide variations caused neither by neoPRFs nor anchor residue changes with low predicted affinity (150–500 nM). Each type of neoantigen had a pre-defined weight as follows:

$${\text{Neoantigen}}\;{\text{Type}}\;{\text{Weight}}\,\left( {\text{p}} \right) = \left\{ {\begin{array}{*{20}l} {1, class 1;} \hfill \\ {0.6, class2;} \hfill \\ {0.5, class3; } \hfill \\ {0.25, class4;} \hfill \\ {0.15, class5 } \hfill \\ {0.125, class6} \hfill \\ \end{array} } \right.$$

We then combined these measures using a deep learning-based model to score and rank the neoantigens.

$$\begin{aligned} & {\text{DeepLearningWeight}}\left( {\text{p}} \right) \\ & \quad = \left\{ {\begin{array}{*{20}l} {1, \quad if\; peptide\; identified\; both \;by\;netMHCpan\;and\;deeplearninig\;methods, TPM > 15 \;and\;MHC\;bindscore < 100} \hfill \\ {0.5, \quad if \;peptide \;identified \;both by\; netMHCpan \;and\; deeplearninig \;methods, TPM > 15\; or\; MHC \;bindscore < 100} \hfill \\ {0.25, \quad if \;peptide\; identified \;both by\; netMHCpan \;and \;deeplearninig \;methods, TPM \left\langle { 15 , MHC\; bindscore } \right\rangle 100, rank\; of \;deeplearning < 30} \hfill \\ {0.125, \quad otherwise} \hfill \\ \end{array} } \right. \\ \end{aligned}$$

The deep learning-based model was trained on a large mass spectrometry HLA peptide data set from various human tumors using a neural network structure as follows:

1. Training dataset: 8–11mer peptide paired with HLA genotype from published data on 74 patients by mass spectrometry [26] were selected as positive neoantigen. Some random 8–11mer peptides from reference proteome (Uniprot protein database) paired with HLA genotype were used as negative neoantigen;
2. Data process: Peptides were vectorized using a one-hot encoding scheme; Embedding HLA genotypes to vectors;
3. Model architecture: A 256, 74 neurons fully connected neural networks, using relu and sigmoid as activation function. A 74 long embedding vector of HLA type to control the output from fully connected layer from each HLA type of a patient;
4. Training: Split 10% data as validation set. Use binary-crossentropy as loss function to optimize model until the loss function value of validation set stop decreasing.

Finally, we identified neoantigens with VAF > 0.1, MHC binding affinity < 100 nM, TPM > 15, and HLA alleles with no LOH as high confidence neoantigens.

### Validation cohort from published studies

As raw sequencing data was not available, single-nucleotide variants of 13 patients from published articles were collected. Enzyme-linked immune-spot (Elispot) assays were used to mark SNVs as immunogenic. Finally, 1599 assayed single-nucleotide variants from 13 patients were collected from published studies, 19 of which were immunogenic. Neoantigen prediction using the TruNeo pipeline, NetMHCpan [20], MHCflurry, PSSMHCpan, and DeepHLA started from input of annotated mutation list, HLA genotype, and gene-level TPM. Prediction using EDGE was collected from a published article [33].

Recall rate (true positives/19) was used as evaluation criterion of predictive performance.

### Whole exome DNA and RNA sequencing of a lung cancer patient

A 68-year-old patient (patient 01) with squamous cell carcinoma of the lung (SCLC) was enrolled in the study at Shanghai Tenth People’s Hospital in 2018. This study was approved by the Shanghai Tenth People’s Hospital ethics committee and the patient provided written informed consent. A tumor biopsy and peripheral blood samples were collected for whole exome sequencing and transcriptome sequencing to identify mutations and potential neoantigens. PBMCs were also used to conduct the Elispot assay.

DNA and RNA from fresh tumor that were isolated pre-treatment and DNA from paired blood samples were extracted, purified, and hybridized using the Agilent SureSelect Target Enrichment System kit (Qiagen, USA) according to the manufacturer’s instructions, and paired-end multiplex sequencing of samples was performed on the Illumina Novaseq 6000 sequencing platform. The average sequencing depth was 258× in tumor tissue and 126× in paired peripheral blood.

### Evaluating the performance of neoantigen prediction methods by Elispot assays of PBMCs from a cancer patient

To assess whether the multidimensional pipeline performed better than the single-factor methods, we validated the immune responses to neoantigen candidates identified with each method. The peripheral blood cells were obtained from patient 01, and somatic variants identified as described above. We chose two methods to predict neoantigens, the multidimensional TruNeo and open-sourced deep-learning-based MHCflurry methods [10]. Afterwards, the immunogenicity of the 10 top-ranked candidate peptides identified by each software were validated by the Elispot assay [27]

DCs were cultured as previously described [28]. CD8+T cells sorted from PBMCs were stimulated in 24-well cell culture plates with autologous DCs pulsed with individual neoantigen peptides (10 μg/mL) and IL-7 (10 ng/mL; PeproTech). On day 3, IL-2 (5 μg/mL; PeproTech) was added. Half of the medium was changed, and the addition of cytokines was performed every 3 days, as described previously [2]. After 10 days, the IFN-γ response of the prestimulated T-cells was tested against neoantigens by Elispot assays with a Human IFN-γ Elispot kit (MabTech). The Elispot plates were washed five times with PBS. Prestimulated CD8+T cells and DCs pulsed with neoantigen peptides were added to individual wells of the plates and incubated at 37 °C for 18–24 h in the presence of 5% CO_2_. The plates were washed five times with PBS and incubated for 2 h with 100 μL/well anti-human IFN-γ (7-B6-ALP) at 37 °C. Then, the plates were washed five times with PBS and incubated with BCIP/NBT-plus substrate at room temperature. The resulting spots were counted using a computer-assisted Elispot image analyser (Biosys Bioreader 4000), and custom software was designed to detect spots using predetermined criteria based on size, shape, and colorimetric density. The measurement of the spot-size distribution is a built-in function of the software. According to the established guidelines [27], a positive response was defined when the mean of the antigen-stimulated replicates was greater than or equal to ten spots per well, and the mean of the antigen-stimulated replicates was greater than two times the mean of the replicates of the negative control wells.

True positive rate (true positives/(true positives + false positives)) was used as the evaluation criterion.

### T cell receptor (TCR) sequencing and neoantigen-specific TCR clone analysis

The methods of stimulation and expansion of neoantigen-specific T cells were the same as the methods used for the preparation of prestimulated CD8+T cells. CD8+T cells stimulated by DCs without pulsed peptides or CD8+T cells were used as negative controls. Cells were harvested on day 10 and washed twice with PBS. Cultured T-cell pellets were flash-frozen in liquid nitrogen and stored at − 196 °C.

RNA was extracted from flash-frozen peptide-stimulated T cells using TRIzol reagent (Invitrogen. USA). The CDR3 region of the TCRβ chain was amplified by using the iRepertoire multiplex primer set (iRepertoire, Inc), and sequencing was performed using the Illumina 4000 system (Illumina Inc.). Bioinformatic analysis of productive clones was performed to identify antigen-specific expansion using the following criteria: (1) significant expansion (Fisher’s exact test with a Benjamini–Hochberg FDR, *p* < 0.05) compared to that of T cells cultured without peptide, (2) no significant expansion of the relevant clone in any other peptide-stimulated culture, and (3) an odds ratio > 1 (default value).

## Results

### Overview of the TruNeo pipeline from the processing of next-generation sequencing data to the identification of candidate neoantigens

**Fig. 1:**
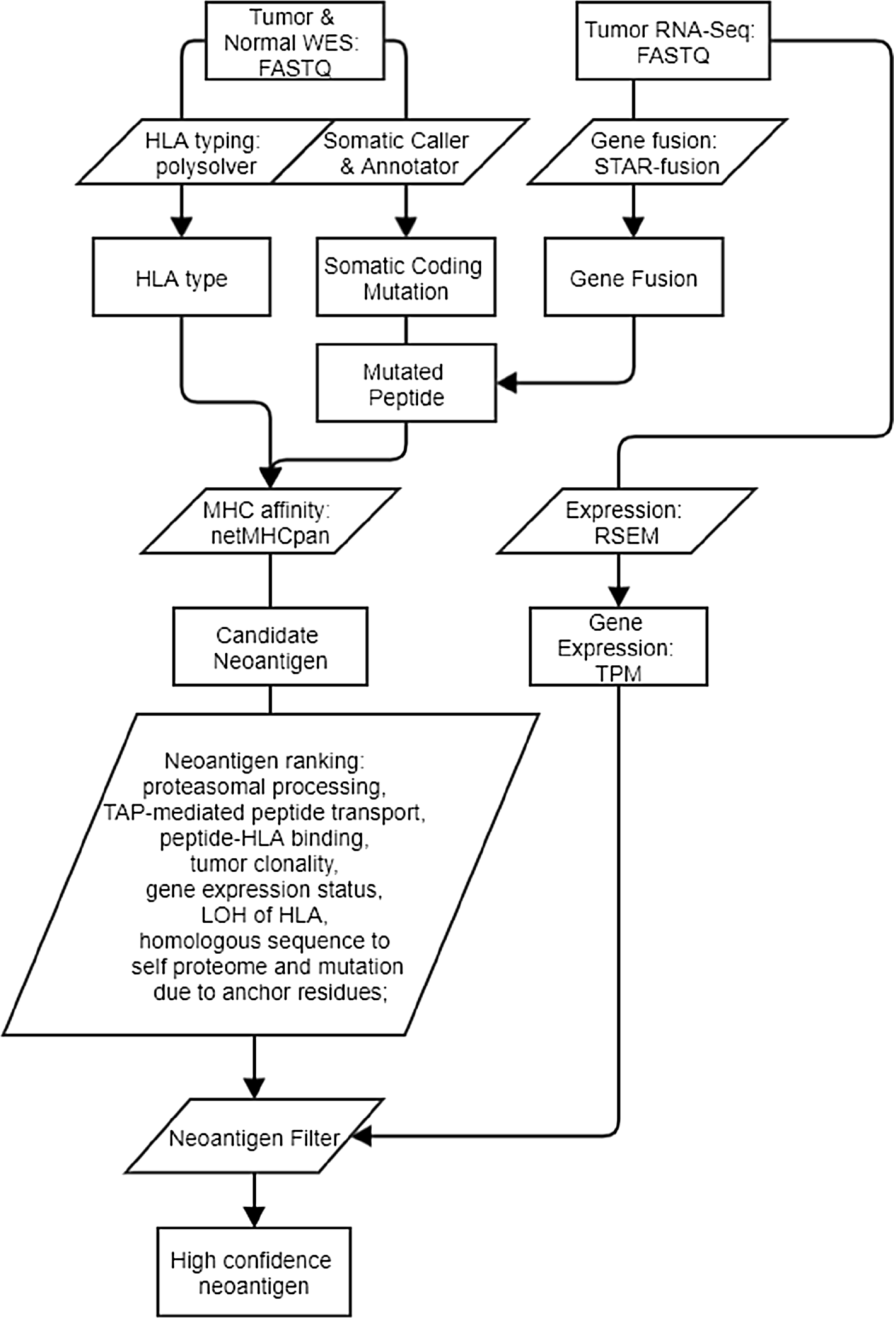
Overview of TruNeo neoantigen prediction pipeline from next-sequencing data to candidate peptides. Flowchart shows the computing steps (trapezoidal box) and corresponding input/result (square box) of TruNeo pipeline. The pipeline includes: (1) Alteration identification, including missense mutations, InDels and gene fusions; (2) HLA typing; (3) gene expression quantification; (4) neo-peptide prediction; (5) MHC I binding affinity prediction for selecting candidate neo-peptide; (6) neo-peptide ranking according to multiple biological processes

In this study, we built an integrated pipeline to predict neoantigens called TruNeo (Fig. 1). The main purpose of TruNeo is to score candidate neoantigen and prioritize 10–20 top-ranked neoantigens for personalized neoantigen-based immunotherapy. The pipeline began with FASTQ data from paired tumor and normal DNA, along with tumor RNA expression data. The first step was to prepare annotated mutation, fusion list, the alleles of human leukocyte antigen (HLA), as well as RNA expression quantification. The second step was to predict candidate neoantigens according to MHC binding affinity (IC50 threshold < 500 nm). All possible 8–11-mer amino acid fragments were derived from SNVs, InDels and fusions, MHC binding affinity was predicted using NetMHCpan. The third step was to integrate a variety of biological factors contributing to immunogenicity for ranking. These factors involved proteasomal cleavage, TAP-mediated peptide transportation, the anchoring residue of HLA, homologous sequences, expression abundance, variant allele frequency and HLA LOH. Candidate peptides were further filtered for high confidence neoantigens based on high affinity of HLA binding, high expression abundance and high VAF. The top-ranked epitopes were considered to be more immunogenic as candidate neoantigens (see “Methods” section).

### The performance of TruNeo and other algorithms on published data

**Fig. 2:**
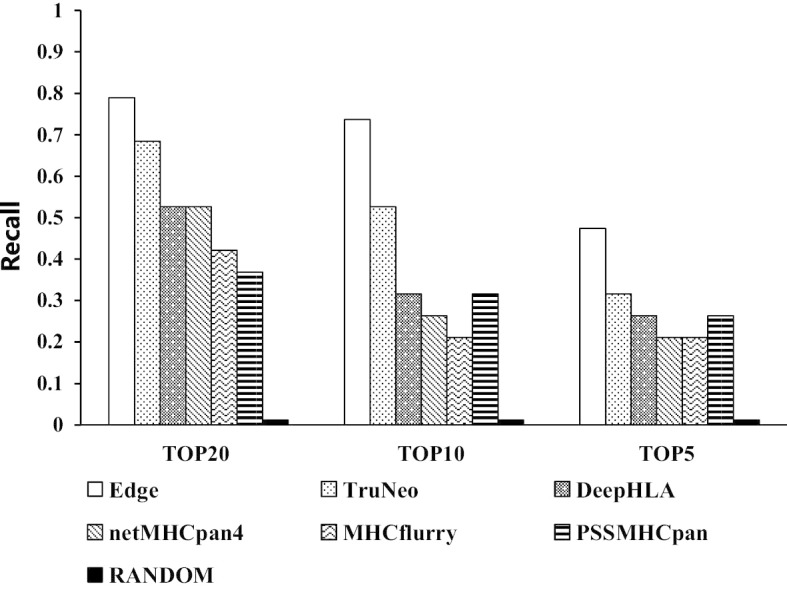
Proportion of immunogenic neoantigens from published literature predicated by TruNeo and other software. 19 out 1599 published mutations were identified with pre-existing T-cell responses. Non-expressed mutations were removed for each software except TruNeo. Neoantigens were ranked by each software with given order. TruNeo could identified more neoantigens than MHCflurry in Top 5, Top 10 and Top 20 level. The RANDOM recall was the expected recall to randomly pick 20, 10, or 5 candidates out of the 1599 mutations, thus 19/1599 = 1.19%

We collected 1599 non-redundant SNVs from 13 patients through published articles [29–31], among which 19 neoantigens were identified as immunogenic true positives that caused a T-cell response using IFN-γ ELISPOT assays. Predictive performance was compared among of TruNeo and other 5 algorithms, including NetMHCpan 4.0, MHCflurry, PSSMHCpan [32], DeepHLA [33] and EDGE [26]. NetMHCpan, MHCflurry, and PSSMHCpan are machine learning models trained on peptide-HLA binding dataset, which identify neoantigen based only on peptide-HLA binding affinity. DeepHLA and EDGE are deep learning models trained on HLA specific tumor mass spectrometry data, which can predict the presented neoantigens or immunogenic neoantigens. To compare the 6 algorithms, the recall rate was calculated in the top ranked 20, 10 and 5 neoantigens provided by each algorithm. Our results (Fig. 2, Additional file 1: Tables S1) showed that TruNeo could rank the immunogenic true positive neoantigens better than HLA binding-based prediction methods (*p* = 0.098, one-sided paired Wilcoxon rank sum test). For example, TruNeo was able to rank 13 immunogenic neoantigens in the top 20, 10 in the top 10 and 6 in the top 5 across 13 patients. In contrast, MHCflurry was able to rank 8 neoantigens in the top 20, 4 in the top 10 and 4 in the top 5. NetMHCpan was able to rank 10 neoantigens in the top 20, 5 in the top 10 and 4 in the top 5. TruNeo also outperformed PSSMHCpan (*p* = 0.021, one-sided paired Wilcoxon rank sum test) and DeepHLA (*p* = 0.039, one-sided paired Wilcoxon rank sum test).

### Performance of TruNeo and MHCflurry in a SCLC patient

**Fig. 3:**
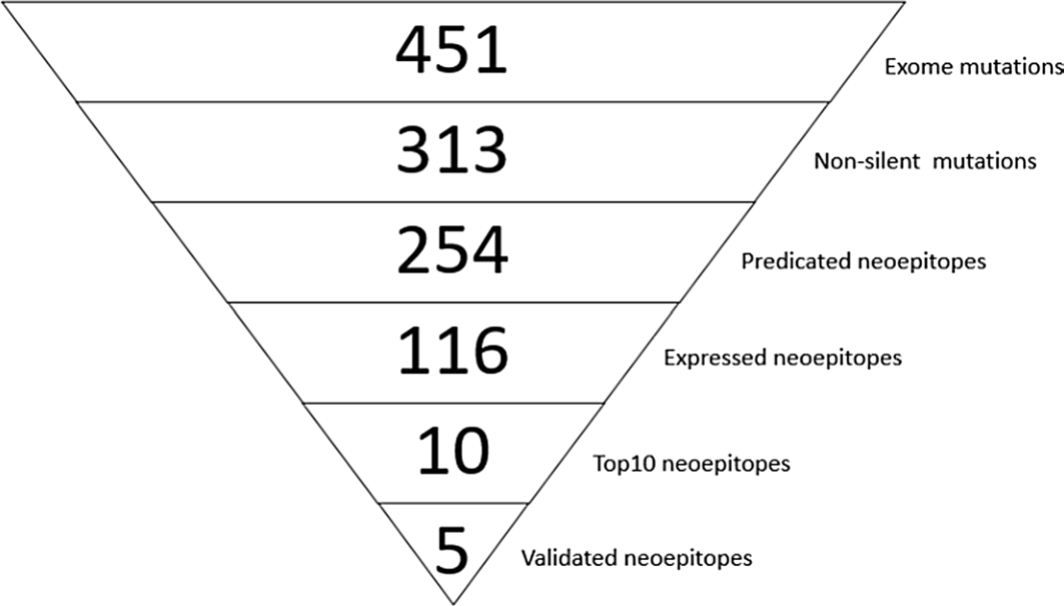
The filtering process of personalized neoantigens for patient 01 by TruNeo software. We applied WES and RNA-Seq sequencing to a patient with advanced squamous cell carcinoma. 451 somatic mutations were detected. 313 of them were non-silent mutations. We predicated 254 neoantigens by TruNeo software. 116 neoantigens could be expressed. Top 10 MHC class I neoantigen were ranked by TruNeo software, and 5 of the top 10 neoantigens were validated by Elispot assay

To further estimate the performance of TruNeo, we predicted the candidate neoantigens in a real case: a patient with advanced squamous cell carcinoma (patient 01). Fresh tumor tissue and blood were collected pre-treatment followed by next generation sequencing. TruNeo were used to call somatic mutations, fusions, HLA genotyping and expression quantification. 451 somatic mutations were identified, including 313 non-silent mutations (Additional file 2: Table S2) (Fig. 3). The non-silent mutations consisted of 297 missense mutations, 2 in-frame mutations, and 14 frameshift mutations. The class-I HLAs of patient 01 were typed as HLA-A*11:01, HLA-A*02:10, HLA-B*40:01, HLA-B*40:01, HLA-C*08:01, and HLA-C*07:02, which were double-checked by assaying tumor and blood samples. These results were further used in neoantigen prediction and ranking through TruNeo and MHCflurry. 254 short candidate peptides (Additional file 2: Table S2) were identified as candidate neoantigens by TruNeo and 395 by MHCflurry. Then, candidate neoantigens were ranked by TruNeo and MHCflurry separately.

**Fig. 4:**
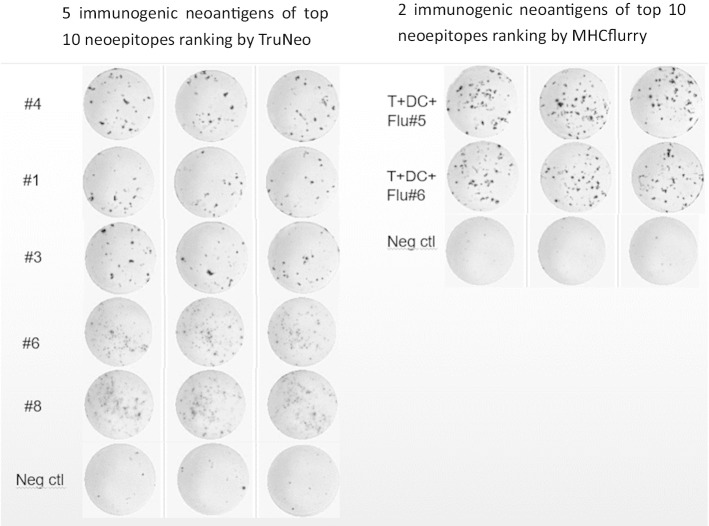
Immunogenic neoantigens of patient 01 predicted by TruNeo and MHCflurry validated by Elispot assays. We have predicted the top10 MHC class I neoantigens for patient01 by TruNeon and MHCflurry. According the rank of the neoantigens were labeled as #1–#10, the immunogenicity of the neoantigen peptides predicted by two models have been validated by the Elispot assay. #1, #3, #4, #6, #8 of the top 10 neoantigen predicted by TruNeo were Elispot positive; #5, #6 of the top 10 neoantigen predicted by MHCflurry were Elispot positive

The top 10 ranked neoantigens selected by TruNeo and MHCflurry separately are shown in Table 1. There was one neoantigen identified by both methods. In total, 19 unique neoantigens peptides were synthetized by GenScript Corporation (Nanjing, China), and then validated using Elispot assays. Five immunogenic neoantigens (#1, #3, #4, #6, and #8) of the top 10 predicted neoantigens of patient 01 identified by TruNeo were showed immunogenic activity, and 2 (#5 and #6) of the top 10 predicted neoantigens identified by MHCflurry were showed immunogenic activity (Fig. 4). The number of true positive neoantigens among the top 10 predicted by TruNeo was 2.5 times higher than that predicted by MHCflurry (50% vs 20%).

**Table 1:** Top 10 neoantigen of a patient01 predicated by TruNeo and MHCflurry

Rank number	TruNeo	Immunogenic validated by Elispot	MHCflurry	Immunogenic validated by Elispot
#1	SEIISFKSL	True	SLFWQTAMV	False
#2	AEVPENVFL	False	LQFEYTFEI	False
#3	SEHGFGPSL	True	LLLCGVQAV	False
#4	VEWLGRCIL	True	ITAEIFMEK	False
#5	QQMGLLTRV	False	ATSPASASK	True
#6	REEKIHDLAL	True	MLICCCCTL	True
#7	LLCKMINLSK	False	ATHPIICFR	False
#8	SSEIISFKSL	True	STVPLDTLK	False
#9	STVPLDTLK	False	LTVETLTKV	False
#10	LEEEINRKM	False	HLEDFLLHI	False

If a neoantigen is immunogenic, there should be specific corresponding TCR clones among PBMCs. To verify the immunogenicity of the neoantigen, we chose the top 1 ranked neoantigen validated by Elispot assays and found that it had two specific corresponding TCR clones (Table 2), which further supports the immunogenicity of this neoantigen from the perspective of the interaction between the peptide and the TCR.

**Table 2:** Significantly expanded TCR clone of #1 neoantigen identified by TCR sequencing (*p* = 0.046, Fisher’s exact test, one-sided)

Clone	Count before stimulation	Count after stimulation	Odds ratio	q value
CAISVGGADNEQFF	6821	500	14.14	< 0.001
CASSYFSEAFF	3092	145	22.06	< 0.001

## Discussion

Recent studies have shown that neoantigen peptides predicted by current bioinformatic tools such as NetMHCpan or MHCflurry were found on the surface of cells was lower than 5% [34, 35], likely because the training data capture information about only one of multiple steps in the HLA class I processing pathway [25]. The pathway from DNA mutations to neoantigens is a complex biological process and the typical pipeline of neoantigen prediction consists of 6 main steps. These steps include identification of somatic mutation, transcription into mutated mRNA, proteasomal processing of the mutated protein, transport of TAP-mediated peptide into the ER lumen, binding of this peptide to the MHC I protein on the endoplasmic reticulum. Finally, the neoepitope MHC complex can be transported to the cell membrane for recognition by TCRs. While current neoantigen identification algorithms rely primarily on the prediction of peptide–HLA binding affinity, it is not sufficient for neoantigen prediction. Secondly current algorithms are mainly machine learning models trained on large in vitro peptide-HLA binding dataset. They have excellent performance as predictors of peptide-HLA binding affinity, but poor performance as predictors of actual neoantigen presentation. Another issue to be considered is tumor cell heterogeneity. Neoantigens may not be expressed in all tumor cells, so the tumor cell fraction and expression abundance of neoantigen should be considered. expression abundance of neoantigen have been proved have relation to the positive rate of neoantigen validation, while current algorithms don’t take into account. Other features, such as mutation due to anchor residues [36], self-proteome homologs, and diversity of HLA molecules have also been shown to be associated with immunogenicity [35]. Thus, the use of a single predictor is less accurate when prioritizing potential neoantigens.

We have demonstrated that TruNeo, a pipeline which considers multiple biological factors, can predict and rank high-quality actionable neoantigens from whole-exome and transcriptome data. TruNeo can predict neoantigens derived not only from point mutations but also from insertions, deletions and fusion genes. Important biological steps, including proteasomal processing and TAP-mediated peptide transport, HLA-binding affinity, presence of homologous sequences, clonality, and gene expression status are considered during annotation and ranking to select the top neoantigens that are most suitable for vaccine development or adoptive cell therapy.

Consideration of features likely contributes to the improved predictive performance of TruNeo compared to existing methods, among which gene expression might be the most critical factor. Thus, the performance of existing methods could be significantly improved by including a gene expression threshold. By raising the minimal TPM (Transcripts Per Million) threshold from 0 to 2, the proportion of CD8-recognized mutations in the top 10 neoantigens was increased from 21 to 42% by MHCflurry, 26–32% by netMHCpan 4.0, 37–47% by DeepHLA, and 37–43% by PSSMHCpan. This result was also demonstrated in previous studies [26, 33], suggesting that expression greatly contributes to high-confidence neoantigen identification.

For class I HLA antigens, we analysed and compared the published data set of the Elispot validation cohort and found that 52.6% of the confirmed positive neoantigens were ranked in the top 10 by TruNeo, which outperformed MHCflurry, NetMHCpan, PSSMHCpan and DeepHLA. For a single case, we found that the positive rate of the top 10 ranked by TruNeo was 50%, compared to 20% using MHCflurry. These results show that the top-ranked neoantigens identified by TruNeo have an increased true positive rate compared with those ranked using standard HLA binding affinity prediction methods.

**Fig. 5:**
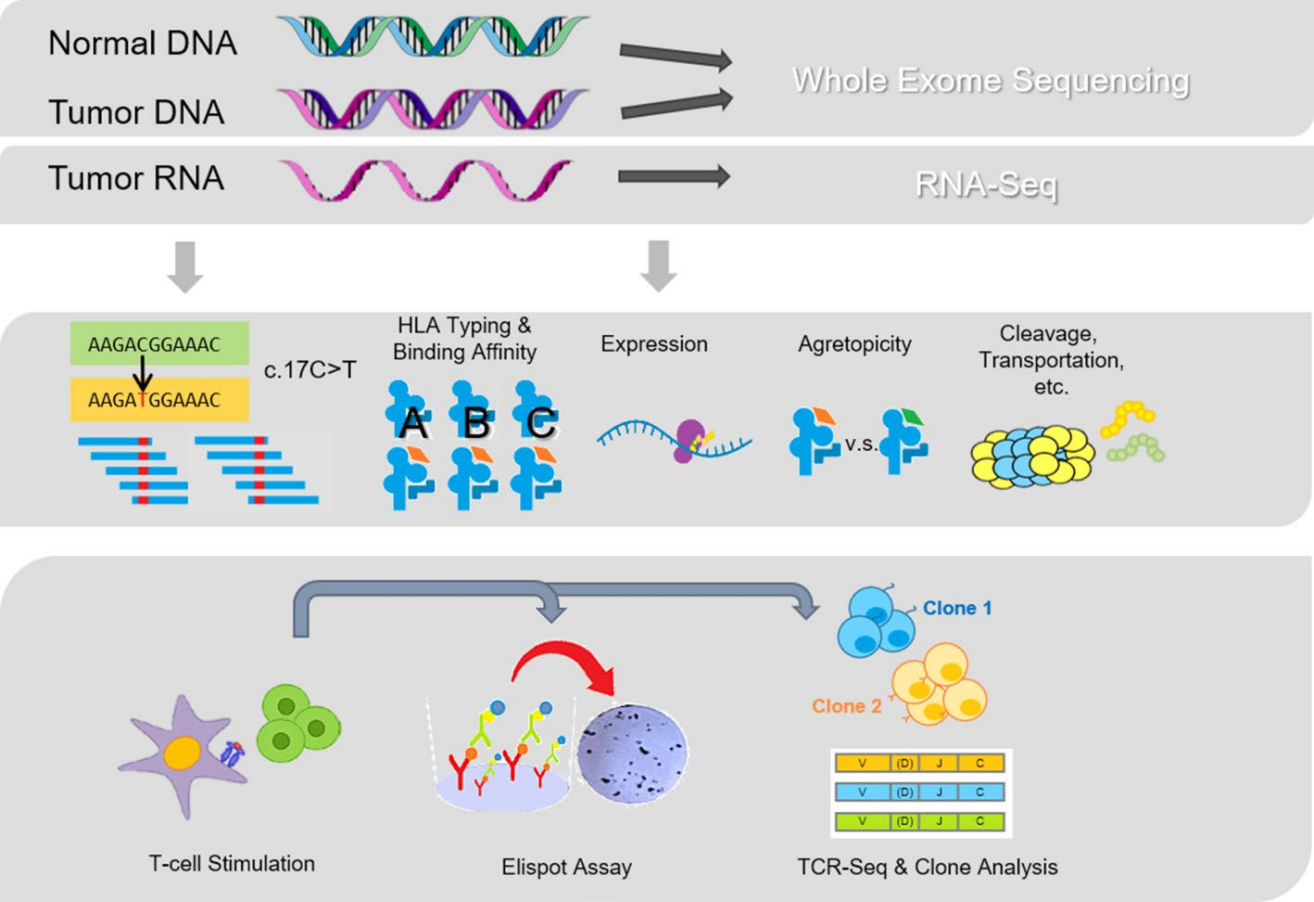
Overview of neoantigen prediction and identification pipeline. To identify the predicted neoantigens, T cells are sorted from PMBC and stimulated by DC pulsed with peptides. Next, CDR3 clones are analysed through TCR Seq, along with applying Elispot assays to confirm immunogenicity

We have not only developed a neoantigen prediction pipeline but also an experimental platform for neoantigen validation (Elispot assay and TCR-seq) (Fig. 5). TCR-seq can provide the TCR CDR3 sequences of immunogenic neoantigens validated by Elispot assays, which are useful in dynamically monitoring the immune function status of patients treated with immunotherapy.

There are also some limitations to our study. As described previously [7], the immunogenicity of 68% of neoantigens was validated post vaccination, and some naive T cells had transformed into neoantigen-specific T cells after vaccination. However, in our study, the PBMCs used for the Elispot assay were collected prior to vaccination, which might influence the true positive rate during validation. Another limitation is that we did not verify HLA class II neoantigens. Two reported clinical studies [2, 7] found that CD4+T cells induced by MHC II comprise the main response by T cells. However, antigenic peptide binding to MHC class II is affected by the long length, poor conservation, and multiple motifs of these antigens, so it is more difficult to predict than binding of MHC class I to antigenic peptides. Work is ongoing to optimize the TruNeo pipeline for prediction of neoantigens presented by HLA class II. A final limitation is that we use a single real patient in comparing TruNeo’s performance to other tools using raw data. Future work should focus on recruiting additional patients to repeat validation experiments for comparing the predictive performance of current tools. This can provide stronger evidence of the relative performance of each method. However, the validation of neoantigen immunogenicity is costly. Many neoantigen peptides must be selected and synthetized for algorithm comparison. Large amounts of peripheral blood is needed for PBMC isolation, or fresh tumor samples are needed to culture TIL for immunogenic validation. Seeking an appropriate cancer patient and blood donor is still a costly and time-consuming work. We hope to improve this aspect with high-throughput, low original input validation platforms in the future.

TruNeo was also compared with other deep learning-based algorithms including EDGE and DeepHLA. We found that EDGE ranked best among the 6 methods. MSIntrinsicEC, another deep learning method, outperformed standard methods by twofold as described [37] which was similar to TruNeo. As described in MSIntrinsicEC, only 16 single-HLA-expressing cell lines were collected for training, which means that MSIntrinsicEC works quite well only with 16 HLA alleles. On the contrary, TruNeo was not limited in terms of HLA alleles. A deep learning method with mass spectrometry data, instead of in vitro HLA–peptide binding affinity data, can deliver neoantigen probabilities without the tediously biological features assessment processes. Deep learning models can be applied to improve TruNeo in the aspect of filtering and ranking when the neoantigen experimental validation data set become large enough. Moreover, neoantigen validated data from spectrometry, Elispot and multiplexed tetramer binding assays are helpful in improving the accuracy of the algorithm.

Cancer immunotherapies which target neoantigens are of growing interest and are in the early stages of human trials, but methods to identify neoantigens require invasive or difficult-to-obtain clinical specimens, require the screening of hundreds or thousands of synthetic peptides or tandem minigenes, or may only be relevant to specific HLA alleles. We have created a neoantigen identification and ranking pipeline that considers multiple factors. Our model increases the immunogenicity rate of the top 10 predicted neoantigens to 50%. We hope that in the future, with the accumulation of positive neoantigen databases that can be used as training sets, developing a prediction method for neoantigens will help to optimize the composition of personalized cancer vaccines and mass spectrometry T cell therapy with high precision and will speed up vaccine and ACT design to meet growing clinical needs.

## Conclusions

TruNeo is a new knowledge-based integrated pipeline that considers multiple factors, including each biological step of HLA presentation, tumor heterogeneity, and HLA-LOH, for the identification and ranking of neoantigens derived from point mutations, insertions, deletions, and fusion genes. The top-ranked neoantigens predicted by TruNeo are highly likely to be immunogenic. The predictive performance of TruNeo and MHCflurry was compared through data from published literature and a single patient. Both sets of data showed that TruNeo exhibited greater performance than MHCflurry. Thus, TruNeo has the potential to advance research on next-generation cancer immunotherapies and improve the efficacy of such targeted treatments.

## Supplementary information

**Additional file 1**. Median rank of epitopes with pre-existing CD8 immune response.

**Additional file 2**. The patient 01’s somatic mutations and neoantigens predicted by TruNeo and MHC flurry.

## Data Availability

The datasets generated and/or analysed during the current study are available at https://www.nature.com/articles/nbt.4313#Sec33 supplementary data 3a.

## Abbreviations

HLA: Human leukocyte antigen
HLA-LOH: HLA loss of heterozygosity
Elispot: Enzyme-linked immune-spot
PBMC: Peripheral blood mononuclear cell
CCF: Cancer cell fraction
neoORFs: Novel open reading frames

## References

[1] Schumacher TN, Schreiber RD (2015). Neoantigens in cancer immunotherapy. Science.

[2] Ott PA (2017). An immunogenic personal neoantigen vaccine for patients with melanoma. Nature.

[3] Hilf N (2018). Actively personalized vaccination trial for newly diagnosed glioblastoma. Nature.

[4] Zacharakis N (2018). Immune recognition of somatic mutations leading to complete durable regression in metastatic breast cancer. Nat Med.

[5] Keskin DB (2019). Neoantigen vaccine generates intratumoral T cell responses in phase Ib glioblastoma trial. Nature.

[6] Chen F (2019). Neoantigen identification strategies enable personalized immunotherapy in refractory solid tumors. J Clin Invest.

[7] Sahin U (2017). Personalized RNA mutanome vaccines mobilize poly-specific therapeutic immunity against cancer. Nature.

[8] Gubin MM (2014). Checkpoint blockade cancer immunotherapy targets tumour-specific mutant antigens. Nature.

[9] Nielsen M, Andreatta M (2016). NetMHCpan-3.0; improved prediction of binding to MHC class I molecules integrating information from multiple receptor and peptide length datasets. Genome Med.

[10] O'Donnell TJ (2018). MHCflurry: open-source class I MHC binding affinity prediction. Cell Syst.

[11] Chabanon RM (2016). Mutational landscape and sensitivity to immune checkpoint blockers. Clin Cancer Res.

[12] Hilf N (2019). Actively personalized vaccination trial for newly diagnosed glioblastoma. Nature.

[13] Li H, Durbin R (2009). Fast and accurate short read alignment with Burrows–Wheeler transform. Bioinformatics.

[14] McKenna A (2010). The Genome Analysis Toolkit: a MapReduce framework for analyzing next-generation DNA sequencing data. Genome Res.

[15] Koboldt DC (2012). VarScan 2: somatic mutation and copy number alteration discovery in cancer by exome sequencing. Genome Res.

[16] Cingolani P (2012). A program for annotating and predicting the effects of single nucleotide polymorphisms, SnpEff: SNPs in the genome of *Drosophila melanogaster* strain w1118; iso-2; iso-3. Fly (Austin).

[17] Matey-Hernandez ML, Brunak S, Izarzugaza JMG (2018). Benchmarking the HLA typing performance of Polysolver and Optitype in 50 Danish parental trios. BMC Bioinform.

[18] McGranahan N (2017). Allele-specific HLA loss and immune escape in lung cancer evolution. Cell.

[19] Lin YF (2013). A combination of improved differential and global RNA-seq reveals pervasive transcription initiation and events in all stages of the life-cycle of functional RNAs in Propionibacterium acnes, a major contributor to wide-spread human disease. BMC Genomics.

[20] Li B, Dewey CN (2011). RSEM: accurate transcript quantification from RNA-Seq data with or without a reference genome. BMC Bioinform.

[21] Haas BJ (2019). Accuracy assessment of fusion transcript detection via read-mapping and de novo fusion transcript assembly-based methods. Genome Biol.

[22] Jurtz V (2017). NetMHCpan-4.0: improved peptide-MHC class I interaction predictions integrating eluted ligand and peptide binding affinity data. J Immunol.

[23] Kandoth C (2013). Mutational landscape and significance across 12 major cancer types. Nature.

[24] Nielsen M (2005). The role of the proteasome in generating cytotoxic T-cell epitopes: insights obtained from improved predictions of proteasomal cleavage. Immunogenetics.

[25] Stranzl T (2010). NetCTLpan: pan-specific MHC class I pathway epitope predictions. Immunogenetics.

[26] Bulik-Sullivan B (2018). Deep learning using tumor HLA peptide mass spectrometry datasets improves neoantigen identification. Nat Biotechnol.

[27] Janetzki S (2015). Guidelines for the automated evaluation of Elispot assays. Nat Protoc.

[28] Dauer M (2003). Mature dendritic cells derived from human monocytes within 48 hours: a novel strategy for dendritic cell differentiation from blood precursors. J Immunol.

[29] Gros A (2016). Prospective identification of neoantigen-specific lymphocytes in the peripheral blood of melanoma patients. Nat Med.

[30] Tran E (2015). Immunogenicity of somatic mutations in human gastrointestinal cancers. Science.

[31] Stronen E (2016). Targeting of cancer neoantigens with donor-derived T cell receptor repertoires. Science.

[32] Liu G (2017). PSSMHCpan: a novel PSSM-based software for predicting class I peptide-HLA binding affinity. Gigascience.

[33] Wu J (2019). DeepHLApan: a deep learning approach for neoantigen prediction considering both HLA-peptide binding and immunogenicity. Front Immunol.

[34] Bassani-Sternberg M (2015). Mass spectrometry of human leukocyte antigen class I peptidomes reveals strong effects of protein abundance and turnover on antigen presentation. Mol Cell Proteom.

[35] Yadav M (2014). Predicting immunogenic tumour mutations by combining mass spectrometry and exome sequencing. Nature.

[36] Garcia-Garijo A, Fajardo CA, Gros A (2019). Determinants for neoantigen identification. Front Immunol.

[37] Abelin JG (2017). Mass spectrometry profiling of HLA-associated peptidomes in mono-allelic cells enables more accurate epitope prediction. Immunity.

